# Landing adaptations in individuals with chronic ankle instability

**DOI:** 10.3389/fspor.2025.1746945

**Published:** 2026-01-05

**Authors:** Zhaoyang Yan, Qi Wang, Xiaoxue Zhu

**Affiliations:** 1 School of Special Education and Rehabilitation, Binzhou Medical University, Yantai, China; 2 Sport Science School, Beijing Sport University, Beijing, China; 3 School of Exercise and Health, Shanghai University of Sport, Shanghai, China

**Keywords:** ankle injuries, chronic disease, joint instability, landing strategy, lateral ankle sprain

## Abstract

**Background:**

This study compared the landing strategy on a flip platform between individuals with and without chronic ankle instability (CAI), to provide a biomechanical basis for reducing re-injury risk.

**Methods:**

55 participants with CAI and 55 without CAI were recruited. Each participant landed on a simulated sprain apparatus with the unaffected limb placed on a support platform and the affected limb on a flip platform. Kinematic data were captured using a 12-camera motion analysis system. Independent-samples t-tests were used for statistical analysis.

**Results:**

Individuals with CAI exhibited lower maximum ankle plantarflexion angle [CAI: 27.1° ± 8.1° non-chronic ankle instability (non-CAI): 31.5° ± 8.2°, *p* = 0.010] and higher maximum hip flexion (CAI: 49.2° ± 12.1°, non-CAI: 41.5° ± 14.2°, *p* = 0.004), maximum hip abduction (CAI: 14.7° ± 4.4°, non-CAI: 12.0° ± 5.5°, *p* = 0.009), maximum knee abduction (CAI: 8.8° ± 4.5°, non-CAI: 5.0° ± 3.3°, *p* < 0.001), and maximum foot toe-out (CAI: 18.7° ± 8.6°, non-CAI: 14.3° ± 4.5°, *p* = 0.002) angles.

**Conclusion:**

Individuals with CAI adopt a cautious landing strategy compared to those without CAI.

## Introduction

As the primary load-bearing structure in lower-limb movement, the ankle-foot complex is particularly susceptible to sports injuries, with an incidence rate of approximately 40% (1, 2). Among these injuries, lateral ankle sprains account for 80% (3), moreover, 32%–74% of individuals develop chronic ankle instability (CAI) following an initial sprain (4). CAI is characterized by persistent pain, instability, recurrent injury, and long-term functional deficits (5, 6).This imposes a substantial economic burden, with direct treatment costs averaging between $292–2,268 per person annually (7), furthermore up to 78% of cases potentially progressing to post-traumatic ankle osteoarthritis (8).

Landing is a fundamental component of sports activities and a common cause of lateral ankle sprains (9). During landing, people often cushion impact by adopting a plantar flexion posture in which the forefoot makes contact first (10, 11). In this position, the narrower posterior part of the talus's trochlea enters the ankle joint cavity formed by the tibia and fibula, resulting in increased clearance within the joint space and reduced joint stability (12). At this moment, sudden changes in ground reaction force direction or shifts in the body's center of gravity can easily lead to rotation at the ankle joint in the coronal plane (13–15). Combined with the anatomical feature where the lateral malleolus is lower than the medial malleolus (12), this creates a tendency for the ankle joint to invert. Especially, landing on an irregular surface, such as uneven ground or an obstacle, can cause sudden ankle inversion (16, 17). When the inversion angle exceeds the tolerance limit of the tissues surrounding the ankle, it results in a lateral ankle sprain (16).

Although it is known that individuals with CAI exhibit different landing strategies on the stable ground compared to those without CAI—such as lower ankle dorsiflexion angle (18–21), greater knee and hip flexion angle (21–23), greater ankle inversion angle (3)—it remains unclear whether these strategic differences persist during landing on flip platforms, which pose a challenging condition and are more prone to inducing lateral ankle sprains. Filling this gap is crucial because actual lateral ankle sprains are typically triggered by landing on flip platforms (16, 17), characterized by a combined movement of plantarflexion and inversion rather than isolated ankle inversion. However, existing studies mostly opt for flat platforms or single-dimension inversion platforms (24, 25). These paradigms hardly replicate the complex biomechanical scenery of real injuries.

Based on the research gaps described above, this study aims to compare the differences in landing strategies between CAI individuals and non-chronic ankle instability (non-CAI) individuals during landing on flip platform, to identify the landing characteristics in individuals with CAI. Participants were required to land on a simulated sprain apparatus to simulate high-risk scenarios involving ankle inversion and plantar flexion (17). We hypothesize that: (1) when landing on a flip platform, individuals with CAI will exhibit landing strategies that differ from those without CAI, e.g., greater ankle plantarflexion angles and smaller hip flexion angles, (2) the landing strategy adopted by CAI individuals may be associated with a higher risk of lateral ankle sprains than that of non-CAI individuals.

## Methods

### Sample size estimate

An *a priori* power analysis was conducted using G*Power 3.1 (Universität Düsseldorf, Düsseldorf, Germany). Based on a previous study that compared the peak hip abduction angle during landings between individuals with and without CAI (CAI: 10.95° ± 12.93°, non-CAI: 2.31° ± 6.76°, *p* = 0.039, Cohen's d = 0.84) (26), at least 78 participants (39 per group) should be recruited to obtain the *α* level of 0.05 and the statistical power of 0.95.

### Participants

A total of 110 participants (55 with CAI, 55 controls) were recruited from a local university via advertisements and leaflets. Participant demographics are summarized in Table 1. Following International Ankle Consortium guidelines (27), CAI inclusion criteria were: (1) ≥1 severe lateral ankle sprain >1 year prior, causing pain/swelling, restricted daily activities ≥1 day; (2) age 18–25 years (60, 61). (3) ≥2 episodes of ankle “giving way” in the past 6 months; (4) self-reported persistent instability/ functional deficits; (5) Cumberland Ankle Instability Tool (CAIT) score <24. non-CAI participants were matched for sex, age (±3 years), height (±5 cm), and body mass (±5 kg), with no LAS history and CAIT score ≥28. Exclusion criteria for all included: (1) prior lower- extremity fracture/surgery; (2) acute lower-limb injury (e.g., sprain) within 3 months; (3) bilateral CAI; (4) vestibular dysfunction or neurological disorders that affect balance; (5) regular use of medications that impair motor control.

**Table 1:** Basic information of participants.

Variables	CAI group	non-CAI group	*P*
Age (mean ± SD, years)	21.2 ± 1.6	21.1 ± 2.4	0.852
Height (mean ± SD, cm)	176.9 ± 8.3	174.6 ± 7.6	0.135
Body mass (mean ± SD, kg)	72.6 ± 10.1	70.7 ± 11.1	0.070
CAIT score (mean ± SD)	17.0 ± 4.5	28.5 ± 0.8	<0.001
Sex (male/female)	44/11	40/15	0.272

Data are presented as mean ± standard deviation. Independent-samples t tests were used for continuous variables; a chi-square test was used for sex. No significant between-group differences were observed for age, height, body mass, or sex, while the CAI group had a significantly lower CAIT score than the non-CAI group. SD, standard deviation; CAI, chronic ankle instability; CAIT, cumberland ankle instability tool.

### Simulated sprain tests

**Figure 1:**
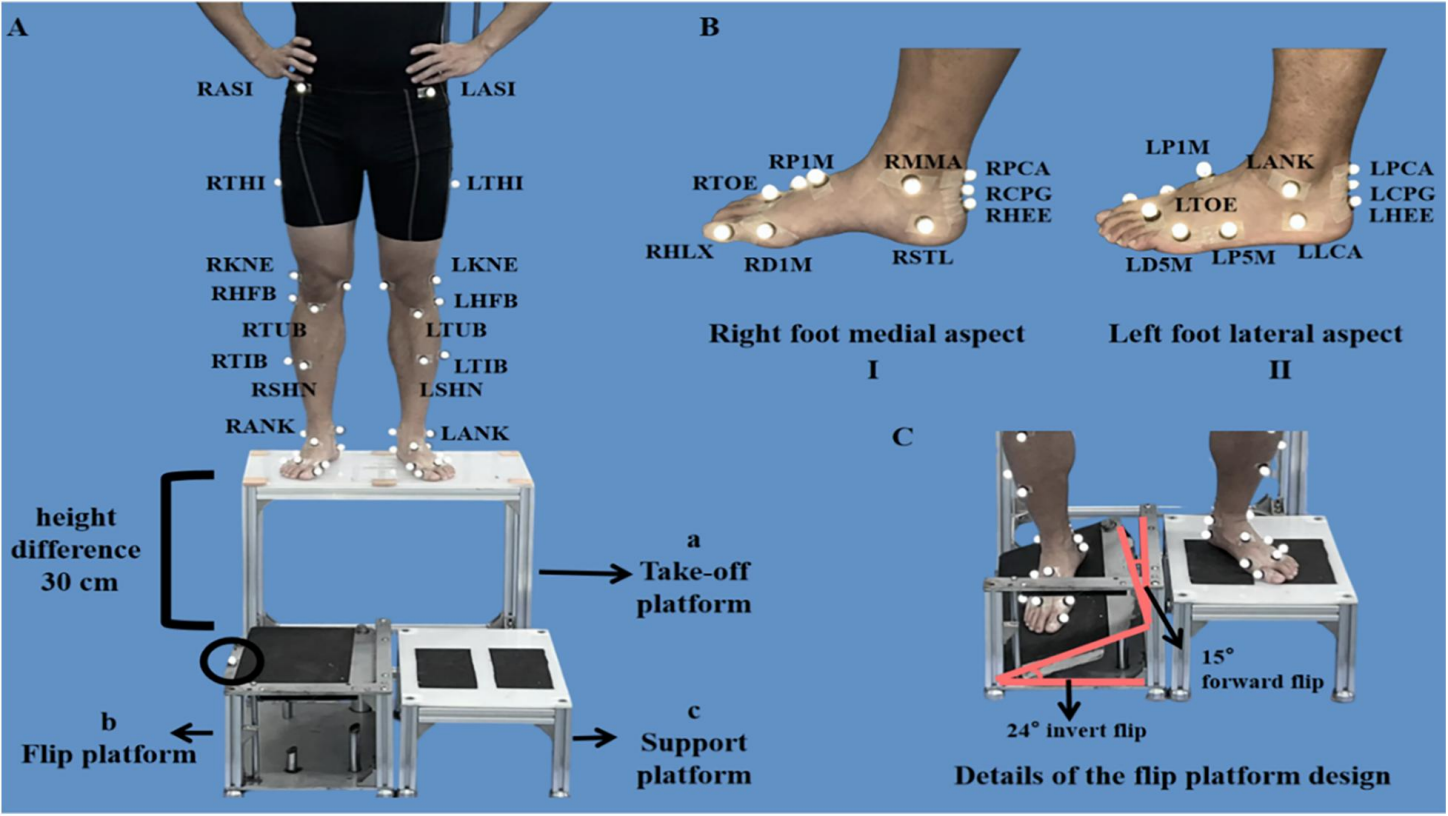
Marker set and simulated inversion-sprain apparatus. **(A,B)** Oxford Foot Model and the simulated inversion-sprain apparatus. **(A,C)** The simulated sprain apparatus. **(A)** Abbreviation key (first letter denotes side; R, right; L, left; remaining letters indicate the anatomical landmark): ASI, anterior superior iliac spine; THI, thigh; KNE, lateral knee; HFB, lateral head of the fibula; TIB, tibial; TUB, tibial tuberosity; SHN, anterior aspect of the shin; ANK, ankle. The simulated sprain apparatus consists of a take-off platform (a), a flip platform (b), and a support platform (c), with a 30 cm height difference between the take-off and landing level. The circled reflective marker on the lateral edge of the flip plate identifies the exact frame of plate collapse. **(B)** TOE, toe; HLX, proximal end of the 1st distal phalanx; DIM, distal medial aspect of the 1st metatarsal; P1M, proximal dorsal aspect of the 1st metatarsal; PCA, posterior calcaneus (proximal); CPG, posterior end of the calcaneus; HEE, heel; LCA, lateral calcaneus; P5M, proximal lateral aspect of the 5th metatarsal; D5M, distal lateral aspect of the 5th metatarsal; MMA, medial malleolus; STL, sustentaculum tali. **(C)** Flip-platform mechanism. When vertical load on the flip plate exceeds the trigger threshold, the plate rotates 24° into inversion and 15° forward (plantarflexion) to reproduce the inversion–plantarflexion posture typical at the onset of a lateral ankle sprain.

Prior to testing, participants changed into standardized tight-fitting shorts and T-shirts. Thirty-six markers were placed on the pelvis, thigh, shank, and foot in accordance with the Oxford Foot Model (Figures 1A,B) (28). Following marker placement, participants completed a 5-min warm-up, after which they performed 15 familiarization trials on the simulated sprain apparatus to experience the perturbation and minimize short-term learning effects (29). Throughout both familiarization and formal testing, participants were secured with an overhead belay harness operated by a researcher to ensure safety (Yunqi Tang et al.2025).

The simulated sprain apparatus (Figures 1A,C) comprised three platforms: the take-off (a), flip (b), and support (c) platforms, with a vertical height difference of 30 cm between the take-off platform and the other two platforms (30). The key component of the apparatus was the flip platform, which would synergistically rotate 24° into inversion and 15° into plantarflexion when detecting the vertical loads exceeding 10 N (31). This specific configuration was designed to simulate the complex motion characteristics of the ankle joint during lateral ankle sprain. A reflective marker affixed to the lateral edge of the flip platform was used to identify the precise frame at which platform collapse occurred.

**Figure 2:**
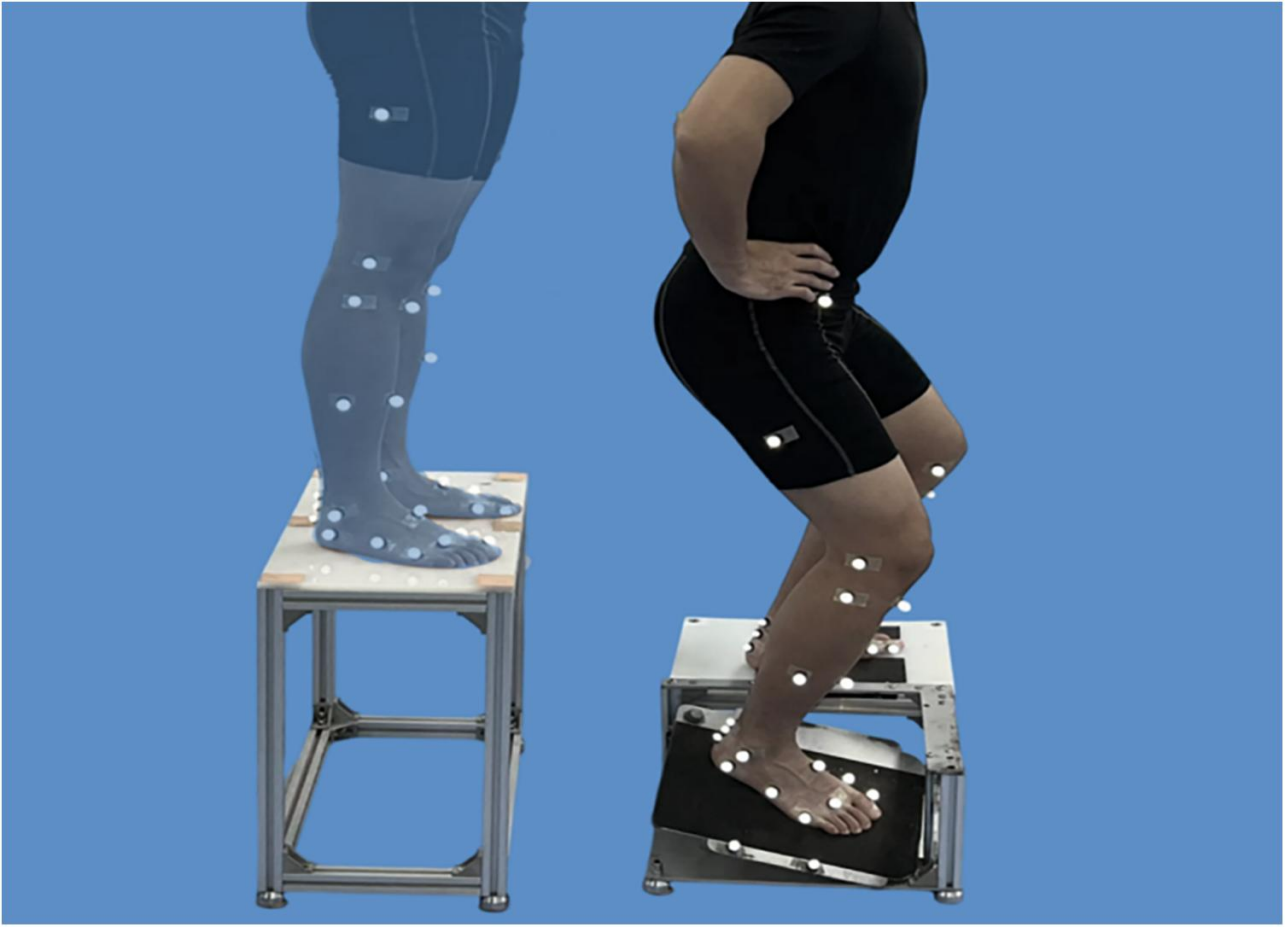
Simulated sprain test. Translucent silhouette (left) shows the start posture on the 30 cm take-off platform with hands on hips. The right panel shows landing on the simulated sprain apparatus: the test limb (affected side in CAI; dominant side in non-CAI) contacts the flip platform, which triggers and rotates 24° into inversion and 15° into plantarflexion upon vertical load; the contralateral limb lands on the support platform (out of frame). Participants self-initiated the step-off after a ready cue and were required to stabilize for ≥3 s after landing.

For each trial, participants stood on the take-off platform with their hands on their hips and eyes directed forward. Upon receiving the “ready” signal from a researcher, they initiated the movement by shifting their center of mass forward until both feet left the take-off platform simultaneously. The test limb landed on the flip platform, while the contralateral limb landed on the support platform (Figure 2). In the CAI group, the affected side was used as the test limb, while in the non-CAI group, the dominant side was used as the test limb, as it is generally considered to represent the functional capacity of both ankles in healthy individuals due to the high degree of symmetry between the two limbs (62).

### Data processing

Kinematic data were collected from the initial contact between the foot and flip platform until 200 ms post-contact (63, 64), which is defined as the landing phase in this study. Initial contact was defined as the time point when the reflective marker on the flip platform's edge descended beyond 1 mm (Figure 2). Recorded marker trajectories were first filtered using a 4th-order Butterworth low-pass filter to eliminate high-frequency noise (65), with a cutoff frequency of 10 Hz (66). The 200 ms time window was then time-normalized to 101 data points to standardize duration across trials and participants. All joint angles analyzed in this study were computed within this normalized time window using Visual3D (v6 Professional; C-Motion, Germantown, MD, USA). Joint angle calculation adopted the Cardan rotation sequence (32).

Maximum ankle plantarflexion angle was defined as the peak negative rotation angle about the medial-lateral axis of the tibia/fibula; maximum ankle inversion angle as the peak positive rotation angle about the floating axis; and maximum ankle external rotation angle as the peak negative rotation angle about the longitudinal axis of the calcaneus (33). Maximum hip flexion angle was defined as the peak positive rotation angle about the pelvic medial-lateral axis; maximum hip abduction angle as the peak negative rotation angle about the floating axis; and maximum hip external rotation angle as the peak negative rotation angle about the femoral longitudinal axis (32). Maximum knee flexion angle was defined as the peak negative rotation angle about the femoral axis; maximum knee abduction angle as the peak negative rotation angle about the floating axis; and maximum knee external rotation angle as the peak negative rotation angle about the tibial longitudinal axis (32). Maximum foot toe-out angle was defined as the peak projection angle in the horizontal plane between the virtual foot's local *y*-axis and the laboratory global *Y*-axis (33).

### Statistical analysis

The normality of each variable within each group was assessed using Shapiro–Wilk tests. Between-group differences (CAI vs. non-CAI) in lower limbs joint angles were evaluated with independent-samples t-tests for normally distributed data or Mann–Whitney *U* tests otherwise. Cohen's d was used to indicate the effect size of *post-hoc* pairwise comparison with the following thresholds: ≤0.20 for trivial, 0.21–0.50 for small, 0.51–0.80 for medium, and >0.81 for large effect sizes (34). Data were presented as mean ± SD, and the significance level was set at 0.05. All analyses were performed in SPSS 26.0 (IBM, Armonk, New York, NY, USA).

## Results

The Shapiro–Wilk test confirmed that all dependent variables were normally distributed.

**Figure 3:**
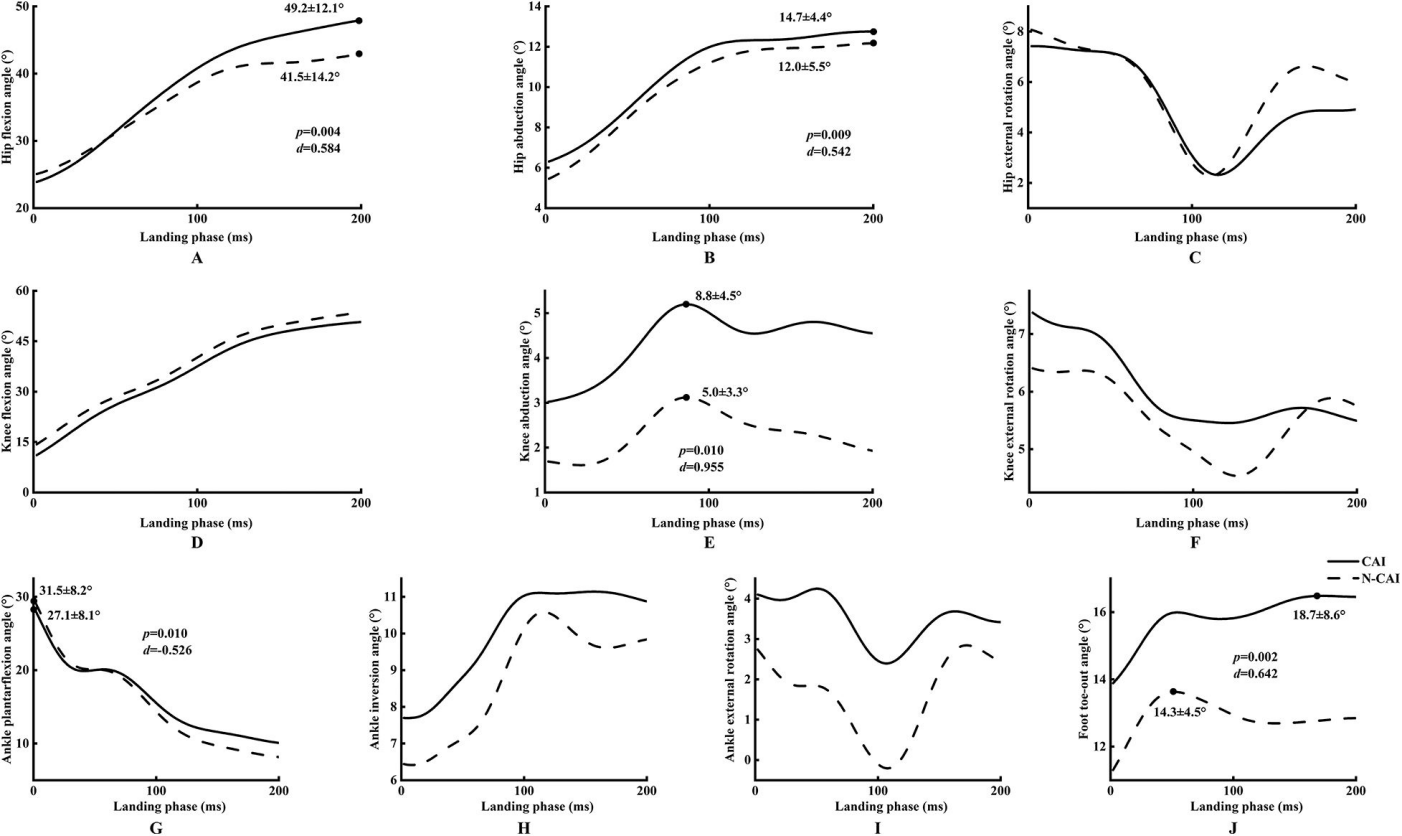
Comparison of lower limbs joints angles between CAI and non-CAI participants. Scatter plots **(A–J)** depict the lower limbs joints angles after contact to 200 ms post-contact; values are mean ± SD. Independent-samples *t* tests were used to compare group differences; *d* quantifies effect size. *p* values indicate statistical significance. CAI, chronic ankle instability; non-CAI, non-chronic ankle instability.

Independent samples *t*-tests showed that, compared with those without CAI, individuals with CAI demonstrated greater hip flexion (CAI: 49.2° ± 12.1°, non-CAI: 41.5° ± 14.2°, *p* = 0.004, Cohen's d = 0.584), hip abduction (CAI: 14.7° ± 4.4°, non-CAI: 12.0° ± 5.5°, *p* = 0.009, Cohen's d = 0.542), knee abduction (CAI: 8.8° ± 4.5°, non-CAI: 5.0° ± 3.3°, *p* < 0.001, Cohen's d = 0.963), foot toe-out (CAI: 18.7° ± 8.6°, non-CAI: 14.3° ± 4.5°, *p* = 0.002, Cohen's d = 0.642) angles, and smaller ankle plantarflexion angle (CAI: 27.1° ± 8.1°, non-CAI: 31.5° ± 8.2°, *p* = 0.010, Cohen's d = 0.526) (Figure 3). To account for multiple comparisons, Bonferroni correction was applied to adjust the significance level.

## Discussion

The primary objective of this study was to compare landing strategies and lateral ankle sprain risk between individuals with and without CAI on flip platform. Hypothesis #1 was rejected, CAI individuals demonstrate smaller ankle plantar flexion angles and larger hip flexion angles. The hypothesis 2 was also rejected, the landing strategies of CAI individuals may reduce lateral ankle sprain risk.

This study showed that compared to individuals without CAI, those with CAI landed with greater hip flexion, smaller ankle plantarflexion angles. This finding is similar to previous research. Previous study found that during landing tasks at different heights, as task difficulty increased, CAI individuals adopted a landing strategy of increasing hip flexion angle and decreasing ankle plantar flexion angle to reduce the risk of lateral ankle sprains (35). We propose that increased hip flexion during landing effectively shifts impact load from the unstable ankle joint to the more stable hip joint, thereby reducing ankle injury risk (11). First, increased hip flexion during landing more activates large hip muscles (such as the gluteus maximus and quadriceps), which possess strong impact absorption capacity during eccentric contraction (36). This effectively disperses external forces, preventing direct impact on the ankle joint (35, 37, 38). Consequently, it reduces the instantaneous load on the ankle, lowering the likelihood of ankle injury (35, 37). Second, a greater hip flexion angle extends the duration for lower limb impact absorption (11). Previous studies indicate that a larger joint range of motion can effectively delay the transmission of impact forces and distribute them across multiple joints and muscles in the lower limbs (11). This reduces the instantaneous high impact force on the ankle joint, further lowering the risk of ankle injury (39). Furthermore, compared to non-CAI individuals, the smaller plantarflexion angle during landing in CAI individuals provides greater joint stability (35), helping the ankle joint more effectively counteract disturbances upon landing. Specifically, a smaller plantar flexion angle increases the contact area between the talars and the ankle fossa formed by the tibiofibular articular surfaces, resulting in a more tightly constructed ankle joint (12). This structural constraint limits the coronal plane rotation of the talus, reducing the tendency for inversion (26) and preventing lateral ankle injuries caused by excessive inversion.

Additionally, we inferred that increased hip flexion may decrease ankle plantarflexion (40). This inference is supported by the classical kinetic chain model proposed by Winter, which emphasizes that in closed-chain tasks, lower limb flexion is a multi-joint coordinated movement, typically coupling hip flexion with ankle dorsiflexion (41). Specifically, hip flexion moves the knee anteriorly, tilting the tibia forward. Due to the ankle's mortise-and-tenon- structure, this anterior tibial tilt shifts the talar dome posteriorly relative to the tibiofibular mortise (42), promoting dorsiflexion and reducing plantarflexion at landing.

This study indicated that individuals with CAI exhibited greater hip and knee abduction and larger foot toe-out angles. These movements observed in individuals with CAI can be explained by a continuous proximal-distal biomechanical chain. During landing, increased hip abduction causes lateral displacement of the femoral head within the acetabulum, thereby elongating the abductor moment arm and inducing outward traction on the distal femur (43). This results in lateral translation of the projection line connecting the foot center of pressure and the body's center of mass relative to the ground. To maintain lower-limb alignment, the tibiofemoral joint undergoes adaptive redistribution of contact forces and load: the tibial plateau shifts laterally relative to the femoral condyles in the coronal plane, manifesting clinically as knee abduction. The anatomical basis for this phenomenon- lies in the differential geometry of the femoral condyles and tibial plateaus: the lateral femoral condyle exhibits a smaller radius of curvature than the medial condyle, while the lateral tibial plateau demonstrates greater flatness compared to its medial counterpart (44). Joint contact forces and load distribution preferentially transfer to the lateral tibiofemoral compartment (45). Additionally, since the lateral meniscus being more mobile than the medial meniscus and can slide along the lateral condyle of the femur (46), enhancing the lateral compartment' capacity for “accommodation” and load adjustment, facilitating knee abduction. This lateral shift in limb alignment propagates distally to the ankle-foot complex. To preserve the mortise configuration between the distal tibiofibular syndesmosis and the talar trochlea, the articular contact zone shifts laterally (47). Subsequent compensatory adjustments occur at the subtalar and transverse tarsal joints to maintain stable plantar contact, resulting in lateral forefoot deviation relative to the hindfoot (48). Collectively, this chain of distal joint displacements is expressed macroscopically as lateral deviation of the foot's long axis relative to the direction of progression—a phenomenon- clinically termed foot “toe-out”.

These findings suggest that continuous proximal-distal biomechanical chain of the hip, knee, and foot can effectively reduce the risk of lateral ankle sprains for the following reasons: First, hip abduction enhances the activation of primary muscles such as the gluteus medius and tensor fasciae latae (49), which are crucial for absorbing and dispersing impact forces (36, 38, 50). These muscles possess significant eccentric contraction capacity, reducing load on the ankle joint and preventing it from sustaining high-impact forces (37), thereby lowering the risk of lateral ankle sprains. Second, foot toe-out, as the distal manifestation of hip and knee abduction, increases the area of the base of support (51), thereby effectively reducing the risk of lateral ankle sprains. Specifically, a larger base of support allows for greater range of center of gravity displacement upon landing (52, 53). This enables individuals to better maintain balance when encountering inversion and plantarflexion disturbances during landing, preventing excessive center of gravity displacement caused by an overly narrow base of support (54). As a result, the risk of lateral ankle sprains is reduced by enhancing balance and control ability. Third, foot toe-out decreases the ankle inversion moment by shortening the lever arm of the ground reaction force relative to the subtalar joint center (55, 56),thereby reducing the inversion load on the ankle and attenuating its sprain risk (57, 58). we propose that toe-out induces medial center of pressure displacement relative to the subtalar joint axis due to foot longitudinal axis deviation (55, 59). This shortens the horizontal distance between the base of support and the subtalar joint inversion/eversion axis (55, 56), reducing inversion moment under equivalent ground reaction force conditions. Consequently, landing-related inversion disturbances decrease, indirectly alleviating the lateral ankle sprains risk during landing. Previous studies support our findings, demonstrating that foot toe-out during landing directly reduces the strain on the anterior talofibular ligament, thereby lowering the risk of lateral ankle sprains (33).

This study has limitations. First, daily activities typically involve shod conditions, where footwear may constrain ankle motion and alter landing biomechanics. In contrast, the current study was conducted barefoot to comply with the marker placement requirements of the Oxford foot model, potentially yielding results that differ from those under shod conditions. Second, due to the flippable character of the simulated sprain apparatus platform, installing a force platform on it presents challenges. Therefore, dynamic data were not calculated in this study. Future studies may consider incorporating dynamic data as needed. Third, there was a significant gender imbalance in both groups. Previous studies have confirmed gender-related differences in biomechanics (67, 68). By matching height and weight between groups, we avoided indirect interference from gender-induced anthropometric differences on biomechanical variables, ensuring results primarily reflect chronic ankle instability's core impact. Future research will optimize recruitment for gender balance, expand the sample size, and use subgroup analysis to explore gender-specific biomechanical differences, enhancing findings' generalizability and relevance.

## Conclusion

Individuals with CAI adopt a cautious landing strategy on a flip platform, demonstrated by greater hip flexion/abduction, knee abduction, larger toe-out angle, and smaller ankle plantarflexion. This strategy may help reduce the risk of lateral ankle sprains during challenging tasks, potentially reflecting an adaptive and cautious response that enhances stability and minimizes injury.

## Data Availability

The raw data supporting the conclusions of this article will be made available by the authors, without undue reservation.
